# Validating the use of bioimpedance spectroscopy for assessment of fluid status in children

**DOI:** 10.1007/s00467-018-3971-x

**Published:** 2018-06-04

**Authors:** Indranil Dasgupta, David Keane, Elizabeth Lindley, Ihab Shaheen, Kay Tyerman, Franz Schaefer, Elke Wühl, Manfred J. Müller, Anja Bosy-Westphal, Hans Fors, Jovanna Dahlgren, Paul Chamney, Peter Wabel, Ulrich Moissl

**Affiliations:** 1 Heartlands Hospital and University of Birmingham, Birmingham, UK; 2 Departments of Renal Medicine and Medical Physics, Leeds Teaching Hospitals NHS Trust, Leeds, UK; 3 Department of Children’s Nephrology, Leeds Teaching Hospitals NHS Trust, Leeds, UK; 4 Pediatric Nephrology Division, Center for Pediatrics and Adolescent Medicine, University of Heidelberg, Heidelberg, Germany; 5 Institute for Human Nutrition and Food Science, Christian-Albrecht University, Kiel, Germany; 6 Universität Hohenheim, Hohenheim, Germany; 7 Department of Pediatrics, Institute of Clinical Sciences, Sahlgrenska Academy at University of Gothenburg, Gothenburg, Sweden; 8 Global R&D, Fresenius Medical Care, Bad Homburg, Germany

**Keywords:** Fluid volume, Bioimpedance, Chronic kidney disease, Overhydration, Total body water, Children, Haemodialysis

## Abstract

**Background:**

Bioimpedance spectroscopy (BIS) with a whole-body model to distinguish excess fluid from major body tissue hydration can provide objective assessment of fluid status. BIS is integrated into the Body Composition Monitor (BCM) and is validated in adults, but not children. This study aimed to (1) assess agreement between BCM-measured total body water (TBW) and a gold standard technique in healthy children, (2) compare TBW_BCM with TBW from Urea Kinetic Modelling (UKM) in haemodialysis children and (3) investigate systematic deviation from zero in measured excess fluid in healthy children across paediatric age range.

**Methods:**

TBW_BCM and excess fluid was determined from standard wrist-to-ankle BCM measurement. TBW_D2O was determined from deuterium concentration decline in serial urine samples over 5 days in healthy children. UKM was used to measure body water in children receiving haemodialysis. Agreement between methods was analysed using paired *t* test and Bland-Altman method comparison.

**Results:**

In 61 healthy children (6–14 years, 32 male), mean TBW_BCM and TBW_D2O were 21.1 ± 5.6 and 20.5 ± 5.8 L respectively. There was good agreement between TBW_BCM and TBW_D2O (*R*^2^ = 0.97). In six haemodialysis children (4–13 years, 4 male), 45 concomitant measurements over 8 months showed good TBW_BCM and TBW_UKM agreement (mean difference − 0.4 L, 2SD = ± 3.0 L). In 634 healthy children (2–17 years, 300 male), BCM-measured overhydration was − 0.1 ± 0.7 L (10–90th percentile − 0.8 to + 0.6 L). There was no correlation between age and OH (*p* = 0.28).

**Conclusions:**

These results suggest BCM can be used in children as young as 2 years to measure normally hydrated weight and assess fluid status.

**Electronic supplementary material:**

The online version of this article (10.1007/s00467-018-3971-x) contains supplementary material, which is available to authorized users.

## Introduction

Fluid management in haemodialysis impacts patient experience, morbidity and mortality [1, 2]. While inadequate fluid removal can lead to oedema and may precipitate heart failure, volume depletion can cause hypotension, dizziness, cramps, abdominal symptoms, prolonged recovery time following haemodialysis and accelerated loss of residual renal function. An increased risk of morbidity and mortality has been associated both with chronic fluid overload [3–5] and with intradialytic hypotension and loss of residual function [6–9].

Determination of optimal fluid status in a dialysis patient is challenging [10]. Conventionally, the assessment of fluid status is based on clinical symptoms and signs. Progressive reduction of target weight until the patient becomes symptomatic is described as ‘probing for dry weight’. Assessment of fluid status is even more challenging in children who often have rapid changes in flesh weight. A number of technologies are now available to aid fluid management in renal patients, of which bioimpedance spectroscopy (BIS) is one of the most widely studied.

Whole-body (wrist-to-ankle) BIS measurements can be used to determine extracellular, intracellular and total body water (ECW, ICW and TBW) volumes [11]. The addition of a 3-compartment body model to distinguish excess extracellular fluid from lean and adipose tissue allows an objective assessment of excess fluid or ‘overhydration’ (OH) [12]. The BIS fluid volume model and the body model are integrated into the Body Composition Monitor (BCM, Fresenius AG, Bad Homburg), which has been well validated [13] and shown to be associated with better survival in adult dialysis patients^,^ [3, 5]. The BCM is increasingly being used to assist the management of adult dialysis patients [14, 15]. For paediatric patients to benefit from this technology, evidence of the applicability of the underlying models in children is required.

The extensive validation of the BCM models against gold standards that has been carried out in adults is impractical in children. However, it is possible to measure TBW in both healthy subjects and haemodialysis patients using child-friendly techniques, against which the TBW estimated by the BCM fluid volume model can be assessed. While there is no gold standard for quantifying excess extracellular fluid, it should be absent in healthy subjects. BCM OH measurements that are close to zero, and without systematic variation with age, in healthy children would provide further confirmation of the validity of the fluid volume model and indicate that the 3-compartment body model developed for adults can be applied to help manage fluid status in children on dialysis.

In this study, we investigated the agreement of BIS-derived TBW (i) with fluid volume obtained by deuterium dilution in healthy children and (ii) with the urea distribution volume (equivalent to TBW) derived from urea kinetic modelling (UKM) in children receiving haemodialysis. A further objective was to check for systematic deviation of the BCM-measured OH from zero in healthy children across the paediatric age range.

## Methods

### Study participants

For the method comparisons, healthy children aged 6 to 14 years related to hospital staff and children receiving haemodialysis at the Paediatric Nephrology Department of Leeds Teaching Hospitals NHS Trust UK were recruited. For the investigation of age-related changes in BCM-measured OH, a large cohort of healthy children aged 2 to 17 years from Germany and Sweden was studied. Children with limb amputations, cardiac or other chronic diseases were excluded.

### Measurement of TBW_BCM and OH

BCM measurements to obtain TBW (TBW_BCM) and OH were made using standard whole-body electrode configuration after the child had been lying down for at least 5 min. TBW_BCM in healthy children was measured once on the dominant side. In children on haemodialysis, measurements were performed on the dominant or non-fistula side before the start of dialysis once a month for up to 8 months.

The BCM measures whole-body impedance over 50 frequencies (from 5 kHz to 1 MHz) and determines extracellular and total body resistance by Cole modelling [16] in order to estimate ECW and ICW using the fluid volume model [11]. The 3-compartment body composition model uses these volumes to separate the body weight into normally hydrated lean tissue mass (LTM), normally hydrated adipose tissue mass (ATM) and excess fluid (or overhydration, OH) [12]. As OH is simply the discrepancy between the actual body weight and the normally hydrated weight (LTM + ATM), it can be positive or negative. OH is typically within a range of − 1.1 to + 1.1 L (10th to 90th percentile) in healthy adults without cardiac or renal complications [14]. However, in both healthy subjects and dialysis patients, the deviation from normal hydration represented by a BCM-measured OH of 1.1L scales with the size of the individual. To compare individuals of different size, OH is normalised to ECW as excess fluid primarily accumulates in the extracellular space.

### Measurement of TBW_D2O

Deuterium oxide (or ‘heavy water’, D_2_O) dilution can provide an accurate measure of TBW [17]. Deuterium is a naturally occurring, stable isotope of hydrogen that is safe for use in children. To avoid the need for infusions or blood samples, the D_2_O was taken orally in a drink containing 1 mL of 7% D_2_O per/kg/body weight (after emptying the bladder) and the children were asked to provide a small (7 mL) urine sample every evening for 6 days starting from the day before taking the D_2_O drink (the baseline). The D_2_O concentration in the urine samples was analysed by isotope ratio mass spectrometry (IRMS) [18] in the Medical Research Council Human Nutrition Research Laboratory in Cambridge, UK. D_2_O distributes throughout the total body water and the initial distribution volume (TBW_D2O) was determined from the mass of D_2_O administered and the decline in the concentration of deuterium in the urine samples [19]. TBW_D2O was calculated using both the multi-point back-extrapolation method and the two-point plateau method for quality control. The volume obtained using the multi-point method was used for analysis.

### Measurement of TBW_UKM

In the haemodialysis cohort, TBW was determined using a modified version of the formal UKM procedure developed by Sargent and Gotch [20]. For each monitored dialysis session, pre- and post-dialysis serum urea samples were taken. The average urea clearance rate for the session was calculated for the dialyser, flow rates and current haematocrit. Urea generation was assumed to be constant and residual renal function was neglected. The urea distribution volume, the ‘kinetic’ V, required to give the measured change in serum urea level (after correction for rebound) with the recorded treatment time and calculated clearance was found by iteration. There were no problems with dialysis delivery such as access recirculation or clotting that would have led to an exaggerated kinetic V in any of the monitored sessions. Like D_2_O, urea distributes throughout the total body water (giving TBW_UKM).

### Funding and ethical approval

The method comparison was funded by a grant from the British Renal Society and the work of one of the main investigator was supported by the National Institute of Health Research (NIHR) Devices for Dignity Healthcare Technology Consortium, UK. The Leeds East Local Research Ethics Committee approved the study protocol and healthy participants were recruited through the hospital’s on-line bulletin board. Parents provided informed consent for the study and children ‘assented’ to take part in line with local recommendations. Data collection for the extended cohort in Germany and Sweden was supported by Fresenius Medical Care, Bad Homburg, by providing the BCM device. Local ethics approval was sought at each site.

### Data analysis

Agreement between methods was assessed using mean and SD (adjusted for multiple measurements when appropriate) of paired differences (Bland-Altman analysis), and *R*^2^ in an *x*/*y* graph. For overhydration, the 10th and 90th percentiles were also reported. Paired *t* tests were used, and a *p* value of < 0.05 was considered to indicate significance.

## Results

Table 1 details age, gender, height, weight and BMI with standard deviation scores of the different groups of healthy children participating in this analysis.

**Table 1:** Subject characteristics stratified by gender and origin [mean ± SD]. BMI is provided in both kg/m^2^ and standard deviation score

Patient cohort	N	Age [years]	Height [cm]	BMI [kg/m^2^]	BMI_SDS
Heidelberg					0.1 ± 1.1
Female	180	9.1 ± 3.8	134.4 ± 21.6	17.7 ± 3.7	
Male	168	9.9 ± 4.5	141.4 ± 28.2	18.4 ± 3.9	
Kiel					0.0 ± 1.0
Female	65	10.5 ± 2.3	144.7 ± 13.4	18.2 ± 3.3	
Male	65	10.5 ± 2.8	146.5 ± 18.1	17.6 ± 3.4	
Gothenburg					0.0 ± 1.0
Female	23	12.0 ± 0.8	158.1 ± 8.0	19.4 ± 4.5	
Male	36	11.8 ± 0.8	155.1 ± 10.5	17.9 ± 2.4	
Fresenius					−0.1 ± 0.2
Female	6	12.8 ± 6.6	146.3 ± 29.8	18.7 ± 2.9	
Male	1	5.0	123	15.3	
Leeds					0.0 ± 0.9
Female	29	10.1 ± 2.5	140.3 ± 14.4	17.8 ± 2.7	
Male	31	10.7 ± 2.3	143.4 ± 15.3	17.1 ± 2.2	

*BMI* body mass index, *SD* standard deviation

### Agreement between D_2_O- and BCM-derived total body water

**Fig. 1:**
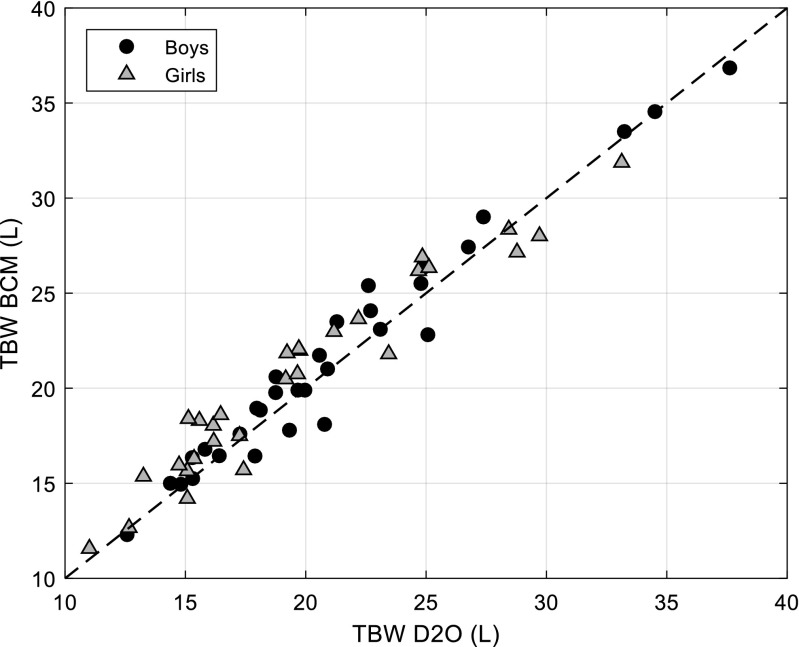
TBW BCM vs. TBW D20 in 60 healthy children aged between 6 and 14 years. Corresponding Bland-Altman plot is shown in Supplementary Fig. S1. All subjects *R*^2^ = 0.97 (*p* < 0.001), Girls *R*^2^ = 0.97 (*p* < 0.001) and Boys: *R*^*2*^ = 0.98 (*p* < 0.001). *BCM* Body Composition Monitor, *D*_*2*_*O* deuterium oxide, *TBW* total body water

Sixty-one healthy children received TBW assessments by D_2_O dilution. Sixty children (28 female, median age 10.3) were able to provide sufficient urine samples for analysis. TBW_BCM was calculated using unadjusted model as provided by BCM Version 3.2 and above. The mean TBW_BCM (±SD) was 21.1 ± 5.6 L and that by deuterium dilution was 20.5 ± 5.8 L. There was good agreement between TBW_BCM and TBW_D2O (*R*^2^ = 0.97, Fig. 1) with a bias of + 0.6 L and 95% limits of − 2.0 to +3.2 L (Fig. S1).

### Agreement between BCM- and UKM-derived total body water in children receiving haemodialysis

**Fig. 2:**
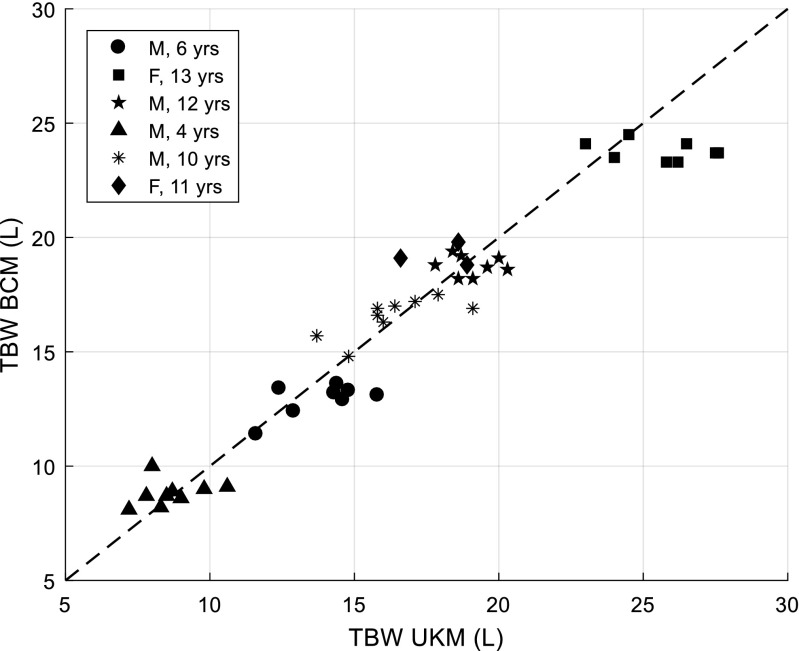
TBW_BCM vs. TBW_UKM in 6 children on haemodialysis. Measurements were made over 8 months. Corresponding Bland-Altman plot is shown in Supplementary Fig. S2. The same data but individually averaged is shown in Supplementary Figs. S3 and S4. Dashed line indicates line of identity. *TBW* total body water, *BCM* Body Composition Monitor, *UKM* Urea Kinetic Modelling

Six children between the ages of 4 and 13 years (2 female, median age 10.5 years) were receiving regular haemodialysis at the time of the study. Table 2 details age, gender, primary renal diagnosis, months on dialysis, height, BMI, requirement for ultrafiltration and months to transplantation for children on haemodialysis participating in the study. Forty-five concomitant measurements of TBW were taken by BCM and UKM over an 8-month period. There was good correlation between TBW_BCM and TBW_UKM (Fig. 2). The mean difference between the methods was − 0.4 L, 2SD = ± 3.0 L (Fig. S2). The mean difference between individually averaged data (*N* = 6) was 0.2 L, range (min to max) = − 1.2 to 1.9 L (Figs. S3 and S4). TBW_BCM showed better precision of the individual monthly measurements with a mean coefficient of variation (CV=SD/mean) of 3.5% compared to 7.7% for TBW_UKM.

**Table 2:** Characteristics of haemodialysis cohort

Patient	Gender	Primary renal diagnosis	Age [years]	Months on HD	Height [cm]	BMI [kg/m^2^]	UF required on HD	Months to tx
1	M	Congenital renal dysplasia	4	38	89	18.9	Yes	8
2	M	Congenital renal dysplasia	6	6	107	20.5	No	14
3	M	Atypical haemolytic uraemic syndrome	10	3	137	14.6	Yes	6
4	F	Congenital renal dysplasia	11	1	139	20.5	No	4
5	M	Nephronophthisis	12	18	140	16.7	Yes	15
6	F	Unknown cause	13	9	154	20.3	Yes	6

*HD* haemodialysis, *BMI* body mass index, *UF* ultrafiltration, *Tx* kidney transplantation

Please note the age, height, BMI and time on HD are at the start of the study

### Investigation of age-related trends in BCM-measured OH in healthy children

**Fig. 3:**
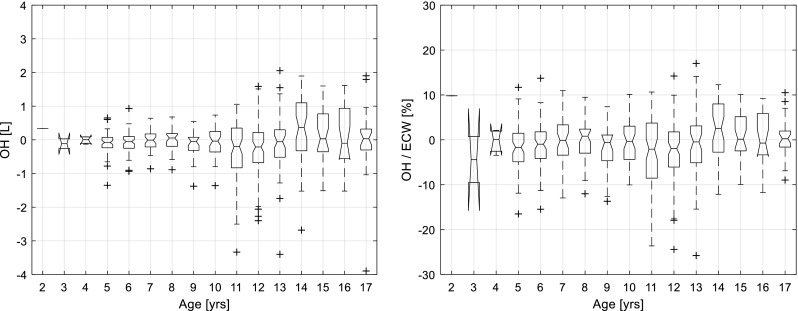
BCM-measured OH (**a**) and OH/ ECW (**b**) in all healthy children (*n* = 634). Boxes indicate interquartile range from 25th to 75th percentile, line is the median, notches indicate 95% confidence interval for median, whiskers are 1.5*interquartile range (covering 99.3% of data assuming normal distribution), crosses = outliers. *BCM* Body Composition Monitor, *OH* over hydration, *ECW* extra cellular water

For all healthy children (*n* = 634) who took part in the method comparison described above, the average BCM-measured OH was − 0.1 ± 0.7 L (10th to 90th percentile − 0.8 to + 0.6 L). The OH normalised to extracellular water (OH/ECW, mean ± SD) was − 1.0 ± 6.3% (10th to 90th percentile − 8.5 to + 6.4%). There was no correlation between age and OH (*p* = 0.28), (Fig. 3). The age distribution of all children under investigation is shown in Fig. S5.

For the subset of healthy children from Leeds (*n* = 60), the BCM-measured OH (mean ± SD) was 0.2 ± 0.5 L (10th to 90th percentile − 0.4 to + 0.9 L). The OH/ECW (mean ± SD) was + 2.3% ± 5.8 (10th to 90th percentile − 5.0 to 9.9%). There was no correlation between age and OH in this subset (*p* = 0.25).

The cohort from Germany and Sweden included 574 healthy children (349 from Heidelberg, 130 from Kiel, 34 from Bad Homburg and 61 from Gothenburg) with a median age of 11 years. Two hundred eighty-six (49.8%) were female. The OH (mean ± SD) was − 0.1 ± 0.7 L which corresponded to − 1.4 ± 6.2% relative to ECW (10th to 90th percentile − 8.6 to 5.9%). Again, there was no systematic variation in OH measured by BCM with age (*p* = 0.28) (Fig. S6).

## Discussion

Bioimpedance measurements are very sensitive to the amount and distribution of fluid in tissue, allowing objective assessment of fluid status. Several bioimpedance-based techniques have been used in studies of children on haemodialysis. Changes in whole-body impedance at 50 kHz reflect changes in body water volume during dialysis [21]. The reactance component of this measurement (Xc) can help identify dry weight [22] and the change in the resistance component (*R*) with fluid removal correlates with intradialytic hypotension and left ventricular mass index [23]. The variability in blood pressure and heart rate during dialysis can be partially explained by vector analysis [24].

Despite its obvious application potential in dialysis, the use of bioimpedance has been largely restricted to research studies due to difficulties in interpreting the data and problems applying predictive equations derived in healthy subjects in patients with abnormal fluid status. The combination of BIS with the 3-compartment body model in the BCM can provide users with a straight-forward assessment of how far a patient is from their normally hydrated weight.

The fluid volume model used to derive ECW and ICW from BIS data includes parameters related to the resistivity of intracellular and extracellular water and body shape, while the 3-compartment model relies on ‘hydration parameters’ (i.e. the proportion of ICW and ECW per kg of lean and adipose tissue). For the BCM to be used in subjects that differ significantly from the reference population, the assumption that the hydration parameters do not require modification needs to be tested [25]. Dialysed children are a particularly critical population in this regard; differences in model parameters in growing and maturing children would be expected to impact the agreement between BCM-measured parameters and measurements using other techniques.

To assess the validity of the fluid model, we compared the TBW estimates by BCM with D_2_O dilution-derived measurements in a cohort of healthy children whose BMI distribution was consistent with that of a large German cohort reported previously [26]. There was good agreement between BCM- and D_2_O-derived TBW readings without any systematic differences related to age, suggesting these findings are likely to be generalisable.

The deuterium dilution method used was not suitable for children on haemodialysis for it required serial urine sampling. Urea kinetic modelling was used to calculate the urea distribution volume as an equivalent to TBW in a cohort of children undergoing haemodialysis. Again, we saw good agreement between the BCM- and UKM-derived TBW estimates without systematic differences across an age range of 4 to 13 years. The repeated measurements showed superior reproducibility of BCM-derived TBW estimates as compared to UKM-derived values, which is not surprising considering the simplicity of bioimpedance measurements as compared to the substantial biological and technical variation associated with a methodology requiring repeated blood sampling and laboratory measurements. The advantage of simplicity was also emphasised in a recent study comparing fluid status in children with nephrotic syndrome by bioimpedance and echocardiography [27].

The validity of the 3-compartment model for fluid overload was assessed in healthy children with an assumed normal state of hydration, i.e. only small differences between actual body weight and the normally hydrated weight (the sum of the normally hydrated lean and adipose tissue) reported by BCM. Such differences will be reported by BCM as ‘overhydration’ since the device is primarily designed to detect and quantify excess fluid in the extracellular compartment. The apparent OH reported by BCM in healthy children was small, without any systematic age-related variation from age 2 to 17 years. Furthermore, the relative overhydration observed in the cohort of healthy children (OH/ECW, mean ± SD) was − 1.0 ± 6.3% (10th to 90th percentile − 8.5 to + 6.4%), showing similar variation to adult healthy controls (10th to 90th percentile − 8 to 8%) [28].

While this study does not provide a rigorous validation of the models used by the BCM to determine body water volumes and normally hydrated tissue mass in children, our findings indicate that the models developed for use in adults can also be applied in children. Further justification for implementing the BCM to help the management of fluid status in paediatric dialysis patients will come with practical experience.

It is important to note that the BCM provides an estimate of a patient’s normally hydrated weight, which is not necessarily identical with the actual target weight due to several confounding factors, including variations in the length of limbs relative to the trunk, body temperature, electrolyte levels and the fluid and electrolyte content of the gut, that vary between measurements. Whereas the use of BCM cannot replace good clinical judgement, it can help prevent potentially harmful adjustments in target weight based on deceptive signs and symptoms. In addition, serial BCM readings allow changes in fluid status to be monitored accurately, provided a consistent measurement protocol is used [29].

The added value of BCM to blood pressure monitoring was impressively documented in a study of 463 dialysis sessions in 23 haemodialysed children [30]. Hypertension was present in 39% of dialysis sessions, of which only 31% were associated with moderate to severe BCM-measured OH. The authors concluded that hypertension is not always related to overhydration, and that use of BCM could avoid inappropriate ‘dry weight probing’ in patients with volume-independent hypertension. Furthermore, a recent study in children attempted to determine the clinical utility of BCM-measured fluid status by comparing it to clinical assessment and cardiovascular indicators. Assessment of fluid status based on clinical assessment was shown to be misleading; BCM measurements correlated with established biomarkers and cardiovascular measures [31].

## Conclusion

Our study suggests that the BCM can be used in children as young as 2 years, at least for measurements of normally hydrated weight and fluid overload. The measurements are non-invasive, well tolerated, inexpensive and easy to perform. If carried out according to a standardised protocol [29], BCM measurements are highly reproducible, allowing changes in lean body mass to be distinguished from changes in fluid status and adjustments in target weight to be made before symptoms occur. Further studies are required to demonstrate the benefits of using BCM in guiding fluid management and improving clinical outcomes in this vulnerable population.

## Electronic supplementary material

ESM 1 (DOCX 105 kb)
