# Supplementary material for: Validating the use of bioimpedance spectroscopy for assessment of fluid status in children
Source: Pediatr Nephrol. 2018 Jun 4;33(9):1601–7. doi: 10.1007/s00467-018-3971-x (PMC6061658; doi:10.1007/s00467-018-3971-x)
Supplement: Supplementary file 1 — (DOCX 105 kb) [file 467_2018_3971_MOESM1_ESM.docx]

supplementary Figures:

Figure s1. Bland altman plot of tbw d2o vs tbw bcm in n=60 children.

Figure s2. Bland-altman plot of tbw_bcm and tbw_ukm in 6 children on dialysis.

Figure S3. Individually averaged TBW BCM vs TBW UKM in 6 children on dialysis.

Figure S4. Bland Altman plot of Individually averaged TBW BCM vs TBW UKM in 6 children on dialysis.

Figure S5. Age distribution of all children (N=634).
